# A Comparison of Surgical Morbidity and Scar Appearance Between Gasless, Transaxillary Endoscopic Thyroidectomy (GTET) and Minimally Invasive Video-Assisted Thyroidectomy (VAT)

**DOI:** 10.1245/s10434-012-2613-y

**Published:** 2012-09-01

**Authors:** Brian Hung-Hin Lang, Kai-Pun Wong

**Affiliations:** 1 Department of Surgery, The University of Hong Kong, Hong Kong SAR, China; 2 Division of Endocrine Surgery, Department of Surgery, Queen Mary Hospital, 102 Pokfulam Road, Pokfulam, Hong Kong SAR, China

## Abstract

**Background:**

The gasless, transaxillary endoscopic thyroidectomy (GTET) and minimally invasive video-assisted thyroidectomy (VAT) are both well-recognized endoscopic thyroid procedures, but how their postoperative outcomes are compared remains unclear. The present study was designed to compare surgical morbidities/complications and scar appearance between GTET and VAT at our institution.

**Methods:**

Of the 141 patients eligible for endoscopic thyroidectomy, 96 (68.1 %) underwent GTET and 45 (31.9 %) underwent VAT. Patient demographics, indications, operative findings, pain scores on days 0 and 1, and surgical morbidities were compared between the two groups. At 6 months after surgery, all patients were asked about their satisfaction on the cosmetic result by giving a score (Patient Satisfaction Score or PSS) and their scar appearance was assessed by the 11 domains in the Patient and Observer Scar Assessment Scale (POSAS).

**Results:**

GTET was associated with a significantly longer operating time (84 vs. 148 min, *p* = 0.005), higher pain scores on days 0 and 1 (2.9 vs. 2.3, *p* = 0.042 and 2.2 vs. 1.7, *p* = 0.033, respectively), overall recurrent laryngeal nerve (RLN) injury (6.3 vs. 0 %, *p* = 0.043), and overall morbidity rates (12.5 vs. 2.2 %, *p* = 0.049) than VAT. The actual individual score for the 11 domains in POSAS and for PSS remained similar between the two groups. They remained similar even when patients with morbidity were excluded.

**Conclusions:**

GTET was a technically more challenging procedure and was associated with longer hospital stay, longer operating time, more immediate pain, and increased overall RLN injury and morbidity than VAT. The 6-month POSAS and PSS were similar between the two procedures.

Since the first report of endoscopic parathyroidectomy, an increasing number of endoscopic thyroidectomy (ET) techniques have been described.^1^^–^ 3 These techniques could be classified as two approaches: the direct and indirect, depending on where the incision(s) are made relative to the neck.^2^,^3^ Of the direct approaches, the minimally invasive video-assisted thyroidectomy (VAT), first described by Miccoli et al.^4^ is the most widely-adopted procedure. It is superior to the conventional open operation in terms of less postoperative pain and better cosmetic results.^5^ Similarly, of the indirect approaches, the gasless, transaxillary endoscopic thyroidectomy (GTET) has better cosmetic benefits than the conventional open operation.^6^^–^ 10 However, despite having similar patient selection criteria, the preferred choice varies in different parts of the world and often is dependent on a combination of the surgeon experience, expertise, and patient preference. The obvious benefit of GTET compared with VAT is no neck scar, but it remains unclear how the two procedures actually compare in terms of their surgical morbidity and cosmetic results.^2^^,^ 3 ^,^ 11 To our knowledge, only one previous study compared the transaxillary approach with VAT and reported that the transaxillary approach had more immediate postoperative pain but higher patient satisfaction.^12^ However, it did not compare the surgical morbidity and cosmetic results between the two procedures. Our study was designed to compare surgical morbidities and scar appearance between GTET and VAT performed in our institution.

## Patients and Methods

Both GTET and VAT were started in 2009, and by 2011, 191 patients underwent GTET or VAT. The selection criteria for either approach were the same, namely age younger than 60 years, no previous neck surgery, benign dominant nodule ≤4.0 cm, and potentially malignant nodule <2.0 cm.^11^ All patients who met the criteria and preferred the ET were given an option of GTET or VAT. During the study period, both procedures were assumed to have similar surgical morbidity. All procedures were performed by one surgeon (BHL). For this study, to minimize the effect of a learning curve associated with GTET and VAT, the first 25 cases of GTET and VAT performed were excluded from analysis. Therefore, 141 patients were included for analysis. Ninety-six (68.1 %) patients underwent GTET, and 45 (31.9 %) underwent VAT. All patients had at least a 6-month follow-up after surgery. Demographics, surgical indications, operative findings, and surgical outcomes were compared between the two groups.

### Surgical Technique

Surgical technique in GTET and VAT has been previously described.^2^^,^ 3 ^,^ 13 For GTET, patients were positioned supine with one arm extended. A 4- to 5-cm axillary skin incision was made, and a subcutaneous flap was raised under direct vision to expose the two arms of the sternocleidomastoid muscle. The strap muscle was lifted from the thyroid capsule. An external retractor was then inserted through the wound and lifted upwards to maintain the working space. An additional 5-mm skin incision was made on the medial side of the chest. A 30°, 10-mm video camera and one 5-mm instrument were inserted through the axillary wound and one other instrument through the chest port. During thyroid dissection, the upper pole was retracted downwards and branches of the superior vessels were individually divided. The lower pole was dissected from the adipose tissue, and the inferior thyroid vein was divided close the thyroid gland. The ipsilateral lobe was then retracted medially, and the perithyroidal tissue was carefully dissected. With careful dissection, the recurrent laryngeal nerve (RLN) was encountered and identified. For the contralateral side, the RLN was identified by anterolateral retraction of the lobe away from the trachea. The resected specimen was retrieved through the axillary wound. A 3-mm suction drain was inserted through the main axillary wound. For VAT, a 2-cm skin incision was made 2 cm above the sternal notch. After blunt dissection, tiny spatulas were inserted to maintain the space between the thyroid gland and strap muscle. A rigid, 5-mm, 30° endoscope was inserted for lighting and magnification. The upper thyroid vessels were generally divided and mobilized endoscopically before the rest of the lobe was retracted superior-medially and delivered through the small wound. The rest of the procedure was performed similar to an open procedure under direct vision by keeping the dissection close to the capsule. No drain was used.

### Outcomes Measured

The weight and size of excised specimen, number of parathyroid glands identified, and perioperative complications were prospectively recorded. The same amount of oral analgesics was prescribed after all procedures. Overall, the surgical morbidity rate was calculated based on the total number of patients with perioperative morbidity divided by the total number of patients undergoing the procedure. In other words, if a patient had more than one morbidity, it would be counted as one. Total operating time was calculated from the time of skin incision to closure. A standard visual analogue score (VAS) was used to assess the severity of postoperative pain using a scale of 0 (“no pain”) to 10 (“worse pain imaginable) on day 0 (approximately 2–4 h after surgery) and day 1 (approximately 18–20 h after surgery). For bilateral thyroid resection, serum calcium and phosphate were regularly measured. Calcium ± vitamin D supplements were prescribed for symptomatic hypocalcaemia or if adjusted calcium <2.00 mmol/L. All patients were encouraged to be discharged on postoperative day 1. In all cases, both vocal cords were examined endoscopically 1 day before and within 1 week after thyroidectomy. Any reduction in cord movement was recorded as paresis. The presence of cord paresis lasting >6 months was regarded as permanent. To calculate RLN injury rates, the number of nerves-at-risk was used as denominator.

### Assessing Scar Appearance and Patient Satisfaction

At 6 months, all patients were interviewed and their scars were inspected and assessed by the Patient and Observer Scar Assessment Scale (POSAS). The POSAS is a complete scar evaluation tool developed by plastic surgeons and consists of two numeric scales: the observer scar assessment scale (OSAS) and the patient scar assessment scale (PSAS).^14^^,^ 15 The OSAS has five domains (vascularization, pigmentation, thickness, relief, and pliability), each graded on a 10-point scale ranging from 1 (normal skin) to 10 (worse scar result). The best summary score has five points, and the worse result has 50 points. The PSAS has six domains on scar assessment (pain, itchiness, color, stiffness, thickness, and irregularity), each graded on a 10-point scale ranging from 1 (normal skin) to 10 (worse scar result). The best summary score has six points, and the worse result has 60 points. During the interview, patients were asked about their satisfaction with their overall cosmetic result and their response was rated from 1 (very satisfied), 2 (satisfied), 3 (unsatisfied), to 4 (very unsatisfied; patient satisfaction score [PSS]). PSS has been used for assessing overall cosmetic satisfaction.^16^

### Statistical Analysis

The statistical analysis was performed using the SPSS (version 18.0, SPSS, Inc., Chicago, IL) software package. The χ^2^ tests and Fisher’s exact tests were used for comparison of dichotomous variables. The Student *t* test and Mann–Whitney *U* test were used to compare continuous variables between groups where appropriate. The Pearson’s correlation test was used to correlate two continuous variables. *P* < 0.05 was considered statistically significant.

## Results

The median age of the cohort was 44 (range, 19–60) years, and 136 (96.5 %) patients were female. The median size of the largest nodule by USG was 2.2 (range, 0.3–4.0) cm, and median follow-up was 24.3 (range, 6.7–40.4) months. Table 1 shows a comparison of baseline patient characteristics between the two groups. The male/female ratio was significantly higher in the VAT group (4/41 vs. 1/95, *p* = 0.036). Five (5.2 %) patients in GTET and two (4.4 %) in the VAT had papillary thyroid carcinoma on fine needle aspiration cytology. In view of tumor size >1 cm, four patients (two in each group) underwent a total thyroidectomy and unilateral central neck dissection endoscopically. All received radioiodine ablation afterwards. One patient in GTET had concomitant excision of a parathyroid adenoma on the same side as the hemithyroidectomy.

**Table 1:** Comparison of demographics, surgical indications, extent of resection, size of dominant nodule, and final pathology between gasless transaxillary endoscopic thyroidectomy (GTET) and video-assisted thyroidectomy (VAT)

Variable	GTET (*n* = 96)	VAT (*n* = 45)	*p* Value
Median age at operation (range)	43 (19–60)	45 (22–60)	0.129^a^
Sex			**0.036**
Male	1 (1)	4 (8.9)	
Female	95 (99)	41 (91.1)	
Surgical indications			0.708
Pressure symptoms	17 (17.7)	12 (26.7)	
Thyrotoxicosis	3 (3.1)	4 (8.9)	
Patient preference	17 (17.7)	4 (8.9)	
Indeterminate FNAC	53 (55.2)	23 (51.1)	
Malignancy	5 (5.2)	2 (4.4)	
Concomitant hyperparathyroidism	1 (1)	0 (0)	
Size of largest nodule on ultrasound (cm)	2.2 (0.3–4)	2.2 (1.5–3)	0.117^a^
Extent of resection			0.846
Unilateral thyroid resection	65 (67.7)	32 (71.1)	
Bilateral thyroid resection	31 (32.3)	13 (28.9)	
Final histopathology			0.853
Nodular hyperplasia	73 (76)	32 (71.1)	
Follicular adenoma	9 (9.4)	4 (8.9)	
Grave’s disease	4 (4.2)	2 (4.4)	
Differentiated thyroid carcinoma	10 (10.4)	7 (15.6)	
Coexisting thyroiditis	8 (8.3)	6 (13.3)	0.364

*FNAC* fine needle aspiration cytology

^a^Student’s *t* test

Table 2 shows a comparison of operative findings between the two groups. Two patients in GTET required open conversion, because one had uncontrolled upper pole bleeding and the other had an unsuccessful skin flap preparation. When these two patients were excluded, the operating time remained significantly shorter in VAT (60 vs. 109 min, *p* < 0.001). The median (range) time for axillary skin flap preparation was six (range, 4–20) min.

**Table 2:** Comparison of excised gland weight, dimensions of excised thyroid lobe, and operative findings between gasless transaxillary endoscopic thyroidectomy (GTET) and video-assisted thyroidectomy (VAT)

Variable	GTET (*n* = 96)	VAT (*n* = 45)	*p* Value
Weight of excised thyroid gland (g)	17.2 (5.5 – 67.1)	17 (4.3–54)	0.849^a^
Length of thyroid lobe (cm)	5 (2.5–8.5)	5 (3.5–9)	0.189^a^
Width of thyroid lobe (cm)	3 (2–5.5)	3 (1.5–5)	0.946^a^
Thickness of thyroid lobe (cm)	2.4 (1–5)	2.3 (1–4)	0.227^a^
Number of parathyroid glands identified in unilateral thyroid resection	2 (0–2)	2 (1–2)	0.564
Number of parathyroid glands identified in bilateral thyroid resection	2 (0–3)	3 (1–4)	0.196
*Total operating time (minutes)*			
Overall	112 (50–245)	60 (23–155)	**<0.001** ^a^
After subtracting time for skin flap preparation	102.5 (40–155)	60 (23–155)	**<0.001** ^a^
Unilateral thyroid resection	92 (50–240)	54.5 (23–77)	**<0.001** ^a^
After subtracting time for skin flap preparation	86 (40–227)	54.5 (23–77)	**<0.001** ^a^
Bilateral thyroid resection	148 (69–245)	84 (69–155)	**0.003** ^a^
After subtracting time for skin flap preparation	132 (61–255)	84 (69–155)	**0.019** ^a^
Open conversion	3 (3.1)	0 (0)	0.551
Blood loss (ml)	20 (10–60)	30 (20–60)	0.567^a^

^a^Student’s *t* test

Bold signifies *p* < 0.05

Table 3 shows a comparison of postoperative outcomes and scar assessment between GTET and VAT. Compared with VAT, the GTET had significantly longer length of hospital stay (2.6 vs. 2.0 days, *p* < 0.001) and higher pain score on days 0 and I (2.9 vs. 2.3, *p* = 0.042 and 2.2 vs. 1.7, *p* = 0.033, respectively). Although the temporary and permanent RLN injury rates were not significantly different, the overall RLN injury rate was significantly higher in GTET (6.3 vs. 0.0 %, *p* = 0.043). The two patients with bleeding/hematoma in GTET had flap bleeding recognized in the recovery room, which was managed by manual compression over the flap area. No reexploration was required. Another two patients in GTET had a small (2–3 mm) perforation in the trachea close to the Berry’s ligament and were managed conservatively. No surgical emphysema was detected postoperatively. One patient in GTET suffered from two surgical morbidities (temporary RLN injury and tracheal injury). The overall morbidity rate of the GTET was significantly higher than that of the VAT (12.5 vs. 2.2 %, *p* = 0.049). The first 30 GTET cases had similar overall morbidity rate as the last 30 GTET cases (5/30 vs. 4/30, *p* = 1.000).

**Table 3:** Comparison of postoperative outcomes between gasless transaxillary endoscopic thyroidectomy (GTET) and video-assisted thyroidectomy (VAT)

Variable	GTET (*n* = 96)	VAT (*n* = 45)	*p* Value
Mean (±SD) hospital stay (days)	2.6 (± 0.8)	2 (± 0)	**<0.001**
Mean (±SD) pain score on day 0	2.94 (± 1.33)	2.31 (± 0.95)	**0.042**
Mean (±SD) pain score on day 1	2.24 (± 0.96)	1.71 (± 1.14)	**0.033**
Surgical complications			
Overall RLN injury^a^	8 (6.3)	0 (0)	**0.043**
Temporary^a^	6 (4.7)	0 (0)	0.102
Permanent^a^	2 (1.6)	0 (0)	0.648
Overall hypoparathyroidism^b^	1 (3.2)	1 (7.7)	0.476
Temporary	1 (3.2)	1 (7.7)	0.476
Permanent	0 (0)	0 (0)	–
Bleeding/hematoma	2 (2.1)	0 (0)	0.329
Tracheal injury	2 (2.1)	0 (0)	0.329
Overall surgical morbidity	12 (12.5)^c^	1 (2.2)	**0.049**

*RLN* recurrent laryngeal nerve

^a^Percentages calculated by dividing the total number of nerves at risk

^b^Bilateral thyroid resection cases only

^c^One patient suffered two different morbidities (temporary RLN injury and tracheal injury)

Bold signifies *p* < 0.05

Table 4 shows a comparison of POSAS and PSS between GTET and VAT. The five domains in the OSAS and six domains in the PSAS and PSS were not significantly different between the two procedures. In terms of the actual individual score, skin pigmentation in the OSAS scored highest, whereas color of the scar in the PSAS scored highest. When patients with morbidity from each group were excluded, the score of the 11 domains for POSAS and PSS remained similar between the two groups. There was a significant direct correlation between PSS and OSAS summary scores (*p* = 0.272, *p* = 0.053) as well as the two of the PSAS domains, namely wound stiffness (*p* = 0.399, *p* = 0.004) and wound thickness (*p* = 0.304, *p* = 0.03).

**Table 4:** Comparison of patient and observer scar assessment scale and patient satisfaction score between gasless transaxillary endoscopic thyroidectomy (GTET) and video-assisted thyroidectomy (VAT)

Variables	GTET (*n* = 96)	VAT (*n* = 45)	*p* Value^a^
POSAS score			
OSAS summary score	8 (5–23)	9 (5–32)	0.787
Vascularization	1 (1–6)	1 (1–7)	0.351
Pigmentation	4 (1–8)	3 (1–7)	0.941
Thickness	1.5 (1–5)	1 (1–4)	0.158
Relief	2 (1–4)	1 (1–8)	0.419
Pliability	1 (1–5)	1 (1–8)	0.26
PSAS summary score	11 (6–33)	10 (6–23)	0.703
Is the scar painful?	1 (1–9)	1 (1–4)	0.238
Is the scar itching?	1 (1–7)	1 (1–5)	0.473
Is the color of scar different?	4 (1–8)	3.5 (1–5)	0.991
Is the scar stiffer?	1 (1–5)	2 (1–4)	0.254
Is the thickness of the scar different?	2 (1–8)	1 (1–5)	0.162
Is the scar irregular?	1 (1–8)	1 (1–3)	0.808
Patient satisfaction score	2.2 (1–4)	2 (1–4)	0.66

*POSAS* patient and observer scar assessment scale; *OSAS* observer scar assessment scale; *PSAS* patient scar assessment scale

^a^Mann–Whitney *U* test

## Discussion

Although GTET and VAT are well-established surgical procedures, a direct comparison of the two has been rare. This may be because most centers would adopt one particular approach as their preferred choice. We adopted two different approaches, because it offered patients a choice. Our study compared the surgical morbidities and scar appearance between GTET and VAT. To avoid the effect of a learning curve associated with each technique, our study excluded the first 25 cases of GTET and VAT. This number of 25 was determined by the data derived from centers with similar case volume as ours.^17^^,^ 18

Considering this was a nonrandomized study comparing two different surgical approaches with selection based on patient preference, most patient parameters were generally well-matched between the two groups. However, almost two-thirds of eligible patients chose GTET over VAT. This might have been because our cohort was relatively young and predominantly female, and so understandingly, most preferred not having a visible neck scar after surgery. Furthermore, before our analysis, both procedures were considered to have similar surgical morbidity. However, our data seemed to suggest that GTET had significantly higher risk of overall RLN injury and surgical morbidity than VAT. Temporary RLN injury accounted for almost half of all surgical morbidities in GTET, whereas no RLN injury was found in the VAT group. However, when one looks at series that routinely performed postoperative laryngoscopic examination, our temporary and overall RLN injury rate actually appeared comparable.^19^^,^ 20 It is possible that the rate of RLN injury might have been underreported when routine laryngoscopic examination was not done. In our opinion, there might be several contributing factors for the higher RLN injury rate. First, GTET is technically more challenging. The significantly prolonged total operating time in the GTET partly reflects this. Furthermore, GTET often requires good open and laparoscopic skills, because the initial skin flap preparation requires good open skills, whereas the subsequent steps are totally endoscopic. On the other hand, VAT is not dissimilar to an open procedure, and so the skills of VAT could be more easily mastered. However, the overall morbidity was not significantly different between the first 30 GTET cases and last 30 GTET cases. The other possible contributing factors were the limitations of the laparoscopic instrument and the small operating space, which often led to collision of instruments. Although some suggested the use of robotic-assisted thyroidectomy to address these limitations, we did not find that in our previous comparison.^11^

The higher pain scores during days 0 and 1 in GTET compared with VAT were consistent to those found in one previous study.^12^ However, given the significantly longer operating time and greater amount of tissue dissection involved in GTET, this was not entirely unexpected. Because GTET is a procedure that requires a large flap dissection, we subtracted the time for flap preparation, but the operating time in GTET was still significantly longer than the VAT. The length of hospital stay in the GTET also was significantly longer than VAT (2.6 vs. 2.0 days), although the actual clinical significance remains unclear, because the majority of our conventional open thyroidectomy get discharged within 24 h.^21^ Compared with our own open thyroidectomy results, the GTET’s surgical morbidity appeared significantly higher.^22^ Furthermore, new risks, such as tracheal perforation or even brachial plexus injury, in GTET are rarely encountered in open thyroidectomy.^23^ In terms of cosmetic results, unlike other studies that compared transaxillary approach with open thyroidectomy and found superior cosmetic results in the transaxillary approach, we were not able to find any significant differences in the 11 domains in POSAS or in PSS between GTET and VAT.^6^^,^ 7 ^,^ 9 ^,^ 24 Even when patients with morbidity were excluded, the PSS remained similar in the two groups. Because PSS assessed the overall cosmetic satisfaction, it would have taken into account the different scar location between GTET and VAT.

Despite these findings, we believe that it is still reasonable to offer patients a choice of the two procedures provided that the higher overall morbidity and similar scar outcome and patient satisfaction at 6 months have been clearly explained preoperatively. Ultimately, the choice depends on how far one is prepared to go for “scarless” in the neck. Our study clearly demonstrated that the major difference between the two procedures was the scar location and not the scar appearance.

Because this was a single surgeon’s comparison, our findings require further validation by other surgeons at different centers or in the setting of a multicenter, prospective study. Furthermore, our case selection was based purely on patient preference for a particular approach, which may pose potential biases influencing patients’ self-reporting scar assessment and satisfaction score. Our series probably represented a center with moderate-volume experience; therefore, our results may not reflect the experience of higher-volume centers. Nevertheless, the increased rate of RLN injury and overall morbidity should be conveyed to patients when deciding on the choice of procedure in the future.

## Conclusions

GTET was a technically more challenging procedure and was associated with longer operating time, longer hospital stay, more immediate pain, higher overall RLN injury, and overall morbidity than VAT. The 6-month scar appearance scored by the POSAS and the 6-month cosmetic satisfaction by PSS were similar between the two procedures.
